# Estrogen-stimulated uropathogenic *E. coli* mediate enhanced neutrophil responses

**DOI:** 10.1038/s41598-024-74863-x

**Published:** 2024-10-03

**Authors:** Carolina Pettersson, Rongrong Wu, Isak Demirel

**Affiliations:** 1 School of Medical Sciences, Örebro University, Campus USÖ, Örebro, 701 82 Sweden; 2 Department of Clinical Research Laboratory, Faculty of Medicine and Health, Örebro University, Örebro, Sweden

**Keywords:** Uropathogenic *Escherichia coli*, Neutrophils, Estrogen, Urinary tract infections, Virulence, Cell biology, Immunology, Microbiology, Urology

## Abstract

Urinary tract infection (UTI) is one of the most common bacterial infections worldwide and the most common cause is uropathogenic *Escherichia coli* (UPEC). Current research is mostly focused on how UPEC affects host factors, whereas the effect of host factors on UPEC is less studied. Our previous studies have shown that estrogen alters UPEC virulence. However, the effect of this altered UPEC virulence on neutrophils is unknown. The aim of the present study was to investigate how the altered UPEC virulence mediated by estrogen modulates neutrophil responses. We found that estradiol-stimulated CFT073 increased neutrophil phagocytosis, NETs formation and intracellular ROS production. We observed that the total ROS production from neutrophils was reduced by estradiol-stimulated CFT073. We also found that estradiol-stimulated CFT073 induced less cytotoxicity in neutrophils. Additionally, we found that several cytokines and chemokines like IL-8, IL-1β, CXCL6, MCP-1 and MCP-4 were increased upon estradiol-stimulated CFT073 infection. In conclusion, this study demonstrates that the estrogen-mediated alterations to UPEC virulence modulates neutrophil responses, most likely in a host-beneficial manner.

## Introduction

Urinary tract infection (UTI) is one of the most common bacterial infections worldwide and may cause life-threatening conditions such as pyelonephritis and urosepsis. The most common cause of UTI is uropathogenic *Escherichia coli*(UPEC), which expresses an array of virulence factors necessary for infecting and colonizing the human host^1,2^. Approximately 50% of women will experience at least one UTI during their lifetime and 25% will have a recurrent UTI within six months^1,2^.

Neutrophils play a central role in the first line defense against UTIs, being the first immune cell to arrive at the site of infection^3^. Neutrophils are directly associated with UPEC burden and possess several antimicrobial traits to restrain and clear infections^4^. Neutrophils use several major mechanisms to eliminate pathogens, including phagocytosis, reactive oxygen species (ROS), antibacterial granular agents and formation of neutrophil extracellular traps (NETs)^5,6^. There is an important distinction between exogenous ROS, which is produced as a response to stimuli such as pathogens, in contrast to intracellular ROS which is a part of the cellular metabolism^6,7^. Mice with impaired neutrophil function are more prone to UTIs and exhibit a reduced capacity to clear the infection^8^. This indicates that neutrophils play a crucial role in eliminating bacteria during a UTI. However, neutrophils are also significantly involved in the tissue damage observed during the infection. This damage is associated with the production of ROS and other cytotoxic substances released by neutrophils in the urinary tract^9^.

As antibiotic resistance is increasing, alternative treatment options against common bacterial infections like UTI are needed^1,10^. Understanding the mechanisms of host-pathogen interactions could provide important clues for new treatment regimens. Currently, much of the research on host-pathogen interactions is dedicated to understanding how pathogens, equipped with their virulence factors, manipulate or evade immune responses to establish infections. However, there is a significant gap in our knowledge regarding the impact of host immune factors, such as cytokines and sex hormones, on the virulence of UPEC through cross-kingdom interactions. Most previous studies on UTI and sex hormones like testosterone and estrogen have focused primarily on their effects on the host and the analysis of risk factors^11^. Estrogen is important for protecting the host against UTIs. It is known that decrease in estrogen levels induce vaginal mucosa atrophy and decreased bladder epithelium proliferation^12,13^. Furthermore, reduced estrogen levels have been associated with lower levels of antimicrobial peptides and lactobacilli^13,14^. We have recently shown that post-menopausal levels of estradiol, but not pre-menopausal levels, significantly enhance the growth and biofilm formation of UPEC. Furthermore, we found that pre-menopausal levels of estradiol significantly reduce UPEC-induced mortality of *C. elegans*^15^. In contrast, we have also shown that testosterone increased UPEC growth, biofilm formation, endotoxin release and increased UPEC-induced mortality of *C. elegans*^16^. These findings highlight the direct influence of estrogen and testosterone on UPEC virulence. However, little is known about how the altered UPEC virulence, mediated by estradiol, affects the interaction between UPEC and neutrophils during a UTI. The aim of this study was to investigate if the altered UPEC virulence, induced by estradiol, could modulate neutrophil responses during an infection.

## Materials and methods

### Bacterial growth

The UPEC-strain CFT073 (American Type Culture Collection (ATCC), Manassas, VA, USA) was isolated from a patient with pyelonephritis and was maintained on tryptic-soy agar (TSA) (Becton Dickinson, Franklin Lakes, NJ, USA). Bacteria was grown in minimal salt solution (MSM, manufactured at the substrate department of Örebro University hospital, Sweden), and incubated for 24 h statically at 37^o^C with or without 17β-estradiol (300pg/ml, E2758, Sigma-Aldrich, St. Louis, MO, USA) prior to all experiments.

### Isolation of neutrophils from venous whole blood

Venous whole blood was collected from healthy donors, between the age of 18 to 65, after informed consent. Neutrophil isolation was performed using density gradient centrifugation of Polymorphprep and Lymphoprep (AXIS-SHIELD PoC AS, Oslo, Norway) in accordance with manufacturers protocol^17^. The neutrophils were infected in RPMI 1640 (Lonza, Basel, Switzerland) complemented with 10% heat inactivated fetal bovine serum (Hi-FBS) during the experiments. Neutrophil viability was evaluated with microscopy prior to experiments. Ethical approval has been granted by the regional ethics review board in Uppsala, Sweden (Dnr 2015/437) and the blood collection was performed in accordance with the Declaration of Helsinki.

### Bacterial stimulation of neutrophils

CFT073 was stimulated with estradiol (300 pg/ml) or DMSO in MSM and grown statically for 24 h at 37 °C. Culturing was followed by 2 washes with PBS to remove free estradiol and DMSO. The DMSO or estradiol stimulated CFT073 were used to stimulate neutrophils at multiplicity of infection (MOI) 1 or MOI 10 at 37^o^C for 1 h, 3–6 h. Supernatants and mRNA were collected and stored at − 80 °C until further analysis.

### Cytokine release and cell viability

IL-1β and IL-8 release from neutrophils were evaluated with enzyme-linked immunosorbent assay (ELISA) using the human ELISA MAX Deluxe set (Biolegend, San Diego, CA, USA) according to manufacturer’s instructions. Lactate dehydrogenase (LDH) release was measured with the CyQUANT LDH Cytotoxicity Assay Kit (Thermo fisher Scientific, MA, USA) according to manufacturer’s protocol to determine cell viability after 3 h and 6 h.

### Olink proteomics

The DMSO or estradiol stimulated CFT073 were used to stimulate neutrophils at MOI 10 for 6 h. Supernatants were collected after infection and centrifuged at 5000 g for 5 min and stored at − 80 °C. Neutrophil supernatants were analyzed using the proximity extension assay (PEA) technology. The Olink inflammation panel (Olink Bioscience AB, Uppsala, Sweden) is based on the PEA technology and enables analysis of 92 inflammation-related proteins. The protein values are reported as % normalized protein expression levels (NPX) of CFT073 stimulation.

### mRNA isolation, cDNA synthesis and real-time qPCR

Total RNA isolation from neutrophils was performed using the E.Z.N.A Plasmid DNA Mini Kit I (OMEGA Bio-Tek, GA, USA) according to manufacturer’s instructions. Nucleic acid concentration was measured with spectrophotometry (Nano-Drop ND-1000, Wilmington, NC, USA) and first strand cDNA was synthesized with the High-Capacity cDNA Reverse Transcription Kit in accordance with manufacturer’s protocol (Thermo fisher Scientific). Real time PCR was conducted using Maxima SYBR Green qPCR Master Mix (Thermo fisher Scientific). Primers (IL-1β, pro-caspase-1, NLRP3, IL-1RA and IL-8) were designed by Origene (MD, USA) and manufactured by Eurofins MWG synthesis GmbH (Ebersburg, Munich). 5 ng of cDNA and 250 nM of each primer was loaded for PCR amplification in a CFX96 Touch Real-Time PCR Detection System (Bio-Rad Laboratories, Hercules, CA, USA). The PCR protocol was conducted as followed: Denaturation at 95^o^C for 10 min followed by 40 cycles of denaturation at 95^o^C for 15s prior to annealing at 60^o^C for 60s. The qPCR reaction was followed by a dissociation curve analysis between 60 and 95 °C. The mRNA expression was analyzed using the comparative Ct (ΔΔCt) method and normalized to the endogenous control GAPDH. Fold difference was calculated as 2^− ΔΔCt 17^.

### Measurement of reactive oxygen species (ROS)

A luminol-horseradish peroxidase (HRP) assay was conducted for measurement of total ROS production. Neutrophils were incubated with luminol (0.1 mg/ml, Sigma-Aldrich) and HRP (4U/ml, Roche, Basel, Switzerland) in KRG buffer with Ca^2+^ for 15 min in 37 °C. Cells were infected with DMSO or estradiol stimulated CFT073 at MOI 1 or MOI 10 for 6 h. The luminescence was measured in a microplate reader Cytation 3 (Biotek Inc., Winooski, VT, United States) every third minute for 6 h. The ROS data is presented as % area under the curve of CFT073.

Intracellular ROS was determined using 2,7-Dichlorodihydrofluoroescin diacetate (H2-DCFDA, Thermo fisher Scientific). Neutrophils were incubated with 10 µM of H2-DCFDA in RPMI for 30 min in room temperature (dark). The neutrophils were then washed with PBS to remove extracellular H2-DCFDA and resuspended in RPMI 1640 (10% Hi-FBS). Neutrophils were stimulated with DMSO or estradiol primed CFT073 at MOI 1 or MOI 10 for 1 h. The fluorescence was measured in a microplate reader Cytation 3 at excitation 485 nm and emission 535 nm after 1 h. The ROS data is presented as % of CFT073.

### Phagocytosis and neutrophil extracellular traps (NETs)

To determine phagocytosis, we measured uptake of CFT073 with flow cytometry. Neutrophils were resuspended in RPMI (10% Hi-FBS) and infected with DMSO or estradiol stimulated CFT073 (carrying an eGFP-plasmid) at MOI 10 for 3 h. The neutrophils were then washed twice in PBS (centrifugation at 300xg 5 min). Prior to acquisition in the flow cytometer, extracellular GFP was quenched with 0.2% trypan blue (Thermo fisher Scientific). Phagocytosis was then evaluated by measuring the mean florescence intensity of the phagocytized CFT073 (eGFP) using the Gallios flow cytometer (Beckman Coulter, CA, USA) with 488 nm laser and FL1 525/40 nm band-pass filter. Kaluza Flow Cytometry Analysis v1.3 (Beckman Coulter) was used for data analysis, as previously described^18^.

Neutrophil extracellular traps (NETs) were quantified with the citrullinated Histone H3 (Clone 11D3) ELISA Kit (Cayman chemical company, Ann Arbor, Michigan, USA) according to manufacturer’s protocol after 6 h of stimulation. Immunofluorescence staining was conducted to illustrate NETs release. Neutrophils were infected for 3 h and then washed and fixed with 4% paraformaldehyde for 30 min followed by incubation with 0.1% Triton-X-100 for 10 min. Blocking was performed with 1% BSA in 0.1% Triton-X100 for 30 min. F-acting was stained with Rhodamine phalloidin (400X, Thermo Fisher Scientific) for 20 min in the dark. DNA was stained with 2.5µM of Sytox green (Thermo fisher Scientific) for 5 min and washed three times with PBS. Slides were airdried and mounted using antifade reagent and were stored in the dark at 4 °C. Images were acquired using an Olympus BX53 fluorescence microscope equipped with an Olympus DP74 camera.

### Statistical analysis

All data are shown as mean ± SEM. The differences between the groups were analysed by unpaired Student’s t-test or by one-way ANOVA followed by Bonferroni multiple testing correction. Results were considered statistically significant at *p* < 0.05. n = number of independent experiments.

## Results

### Estradiol-stimulated CFT073 alters neutrophil phagocytosis and total ROS production

**Fig. 1:**
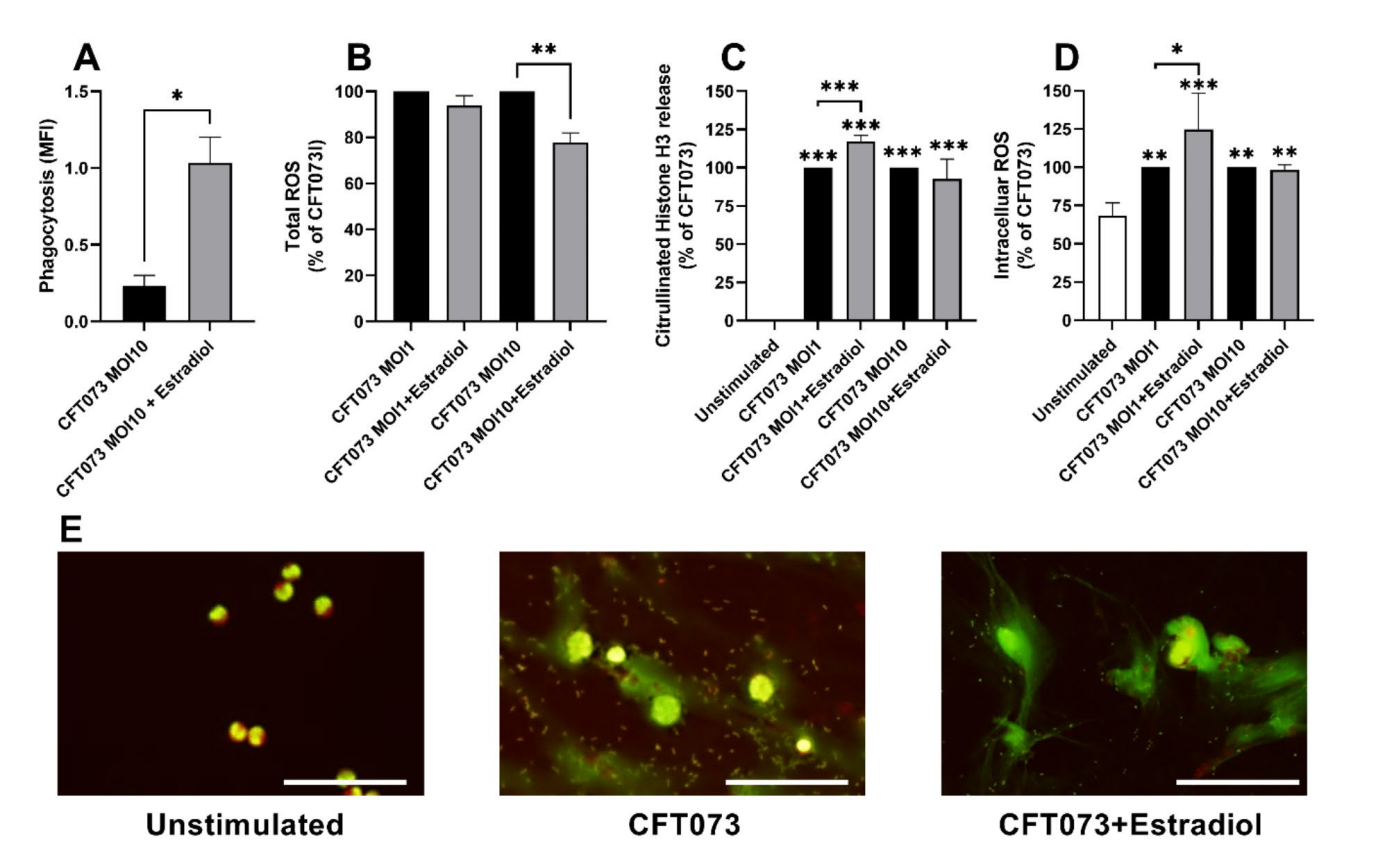
Phagocytosis, total-and intracellular ROS production and neutrophil extracellular traps (NETs). Phagocytosis (**A**), total ROS (**B**), citrullinated histone H3 release (**C**) and intracellular ROS (**D**) were measured in neutrophils after stimulation with CFT073 at MOI 1 or MOI 10 pre-treated with or without estradiol (300pg/ml) for 1 h (**D**), 3 h (**A**) or 6 h (**B**, **C**). NETs formation was visualized with immunofluorescence staining after 3 h (**E**). Scale bar: 50 μm. Data is shown as mean ± SEM, *n =* 4. The asterisk distinguishes statistical significance: *p < 0.05, **p < 0.01, ***p < 0.001.

We started by evaluating if estradiol-stimulated CFT073 could alter neutrophil phagocytosis and total ROS production. We found that estradiol-stimulated CFT073 were significantly more phagocytized by neutrophils compared to unstimulated CFT073 after 3 h (Fig. 1A). We also evaluated total ROS production from neutrophils during 6 h of infection. We found that estradiol-stimulated CFT073 at MOI 10, but not MOI 1, induced significantly less total ROS production compared to unstimulated CFT073 (Fig. 1B).

### Estradiol-stimulated CFT073 induced NETs formation and intracellular ROS

Next, we investigated if estradiol-stimulated CFT073 could alter neutrophil NETs formation and intracellular ROS production. We found that estradiol-stimulated CFT073 (MOI 1) induced significantly increased citrullinated histone H3 release, which is associated with NETs formation, from neutrophils compared to unstimulated CFT073 after 6 h (Fig. 1C). The NETs formation was also validated with fluorescence microscopy (Fig. 1E). In addition, we also found that estradiol-stimulated CFT073 (MOI 1) induced significantly increased intracellular ROS production compared to unstimulated CFT073 after 1 h (Fig. 1D).

### Inflammasome-related mediators are altered by estradiol-stimulated CFT073

**Fig. 2:**
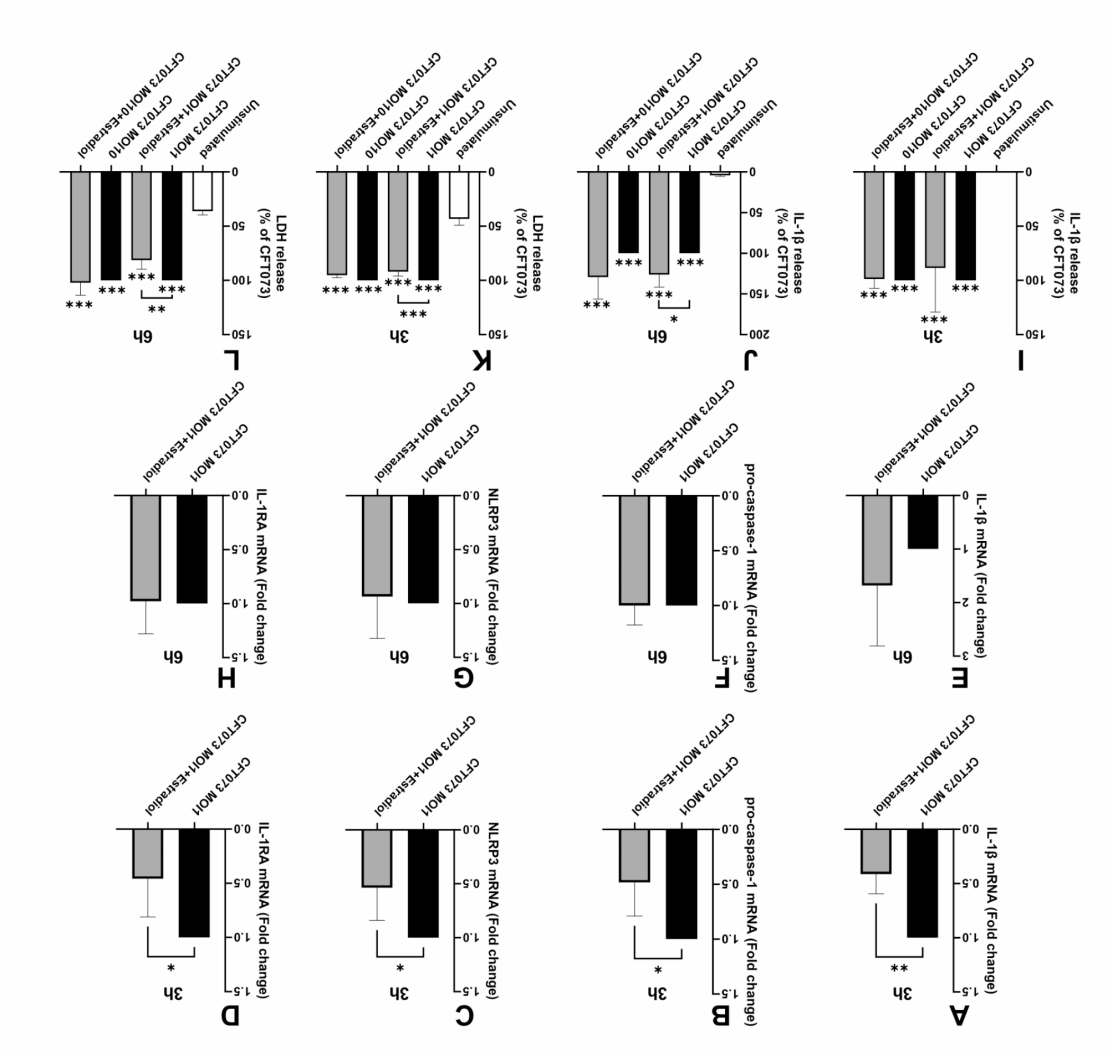
mRNA expression and protein release. Real-time qPCR was performed to measure mRNA expression of IL-1β (**A**, **E**), pro-caspase-1 (**B**, **F**), NLRP3 (**C**, **G**) and IL-1RA (**D**, **H**) from neutrophils after stimulation with CFT073 at MOI 1  pre-treated with or without estradiol (300pg/ml) for 3 h (**A**, **B**, **C**, **D**) or 6 h (**E**, **F**, **G**, **H**). IL-1β (**I**, **J**) and LDH release (**K**, **L**) from neutrophils after stimulation with CFT073 at MOI 1 or MOI 10 pre-treated with or without estradiol (300pg/ml) for 3 h (**I**, **K**) or 6 h (**J**, **L**). Data is shown as mean ± SEM, *n =* 4. The asterisk distinguishes statistical significance: *p < 0.05, **p < 0.01, ***p < 0.001.

We continued to evaluate if estradiol-stimulated CFT073 could alter the expression of inflammasome-related genes and proteins in neutrophils. We found that the mRNA expression of IL-1β (Fig. 2A), pro-Caspase-1 (Fig. 2B), NLRP3 (Fig. 2C), and IL1-RA (Fig. 2D) was significantly decreased after stimulation with estradiol-stimulated CFT073 for 3 h (MOI 1) compared to unstimulated CFT073. However, after 6 h of stimulation, no difference was observed (Fig. 2E-H). Furthermore, we showed that estradiol-stimulated CFT073 and unstimulated CFT073 significantly increased IL-1β release from neutrophils at MOI 1 and 10 after 3 h (Fig. 2I) and 6 h (Fig. 2J) compared to unstimulated neutrophils. Moreover, we observed that estradiol-stimulated CFT073 at MOI 1 induced significantly increased IL-1β release from neutrophils compared to unstimulated CFT073 after 6 h, but not 3 h (Fig. 2I-J). In addition, we found that estradiol-stimulated CFT073 and unstimulated CFT073 significantly increased LDH release from neutrophils at MOI 1 and 10 after 3 h (Fig. 2K) and 6 h (Fig. 2L). However, estradiol-stimulated CFT073 (MOI 1) induced significantly less LDH release from neutrophils compared to unstimulated CFT073 after 3 h (Fig. 2K) and 6 h (Fig. 2L).

### IL-8 expression and release is altered by estradiol-stimulated CFT073

**Fig. 3:**
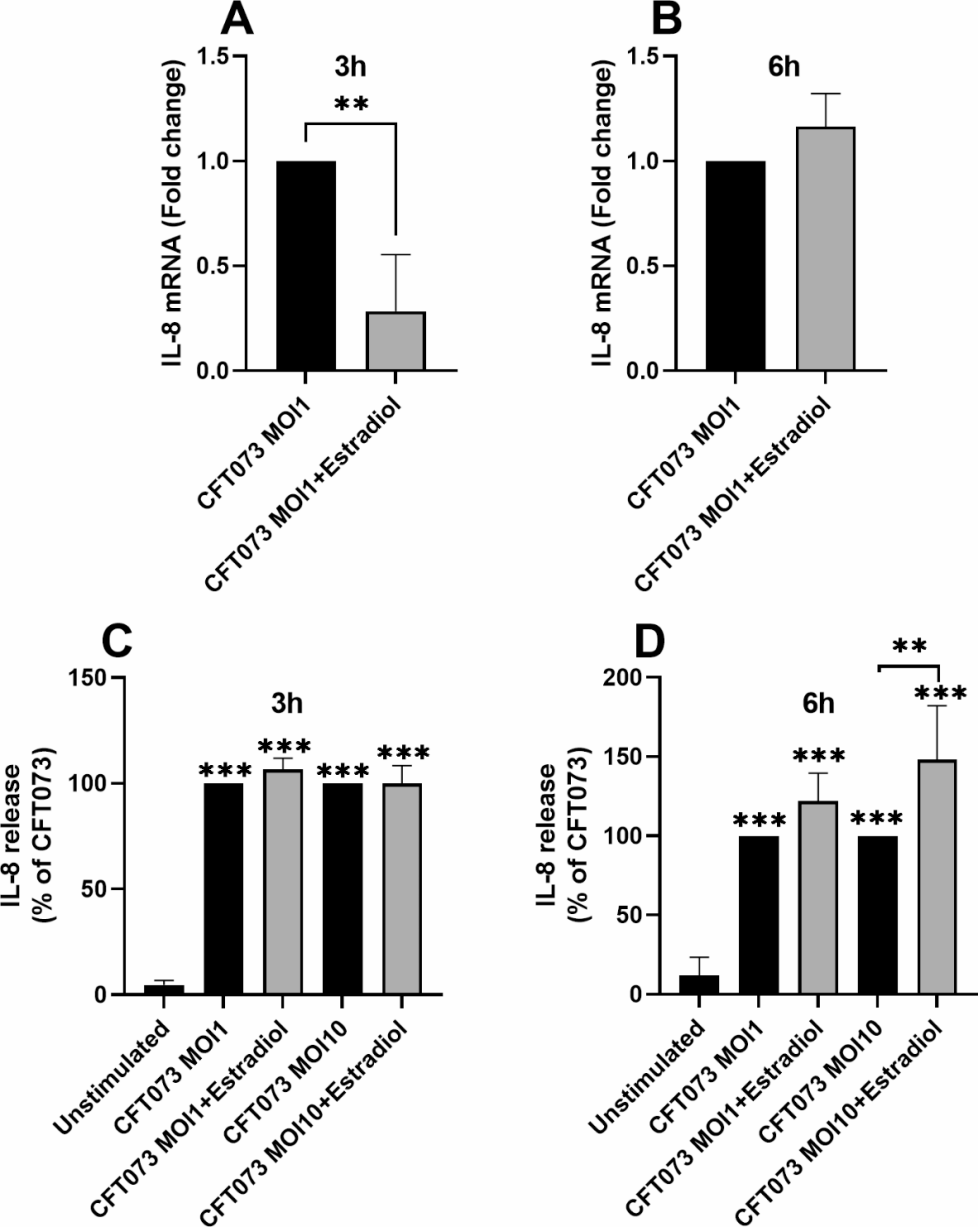
IL-8 mRNA expression and protein release. IL-8 mRNA expression (**A**, **B**) and IL-8 release (**C**, **D**) from neutrophils after stimulation with CFT073 at MOI 1 or MOI 10 pre-treated with or without estradiol (300pg/ml) for 3 h (**A**, **C**) or 6 h (**B**, **D**). Data is shown as mean ± SEM, *n =* 4. The asterisk distinguishes statistical significance: **p < 0.01, ***p < 0.001.

Next, we investigated if estradiol-stimulated CFT073 could alter IL-8 expression and release from neutrophils. We found that that IL-8 mRNA expression was significantly reduced after 3 h (Fig. 3A), but not 6 h (Fig. 3B), by estradiol-stimulated CFT073 compared to unstimulated CFT073. Furthermore, we also found that estradiol-stimulated CFT073 and unstimulated CFT073 significantly increased IL-8 release from neutrophils at MOI 1 and 10 after 3 h (Fig. 3C) and 6 h (Fig. 3D) compared to unstimulated neutrophils. Additionally, we showed that estradiol-stimulated CFT073 (MOI 10) induced significantly increased IL-8 release from neutrophils compared to unstimulated CFT073 after 6 h, but not 3 h (Fig. 3C-D).

### Altered release of inflammatory mediators induced by estradiol-stimulated CFT073

**Fig. 4:**
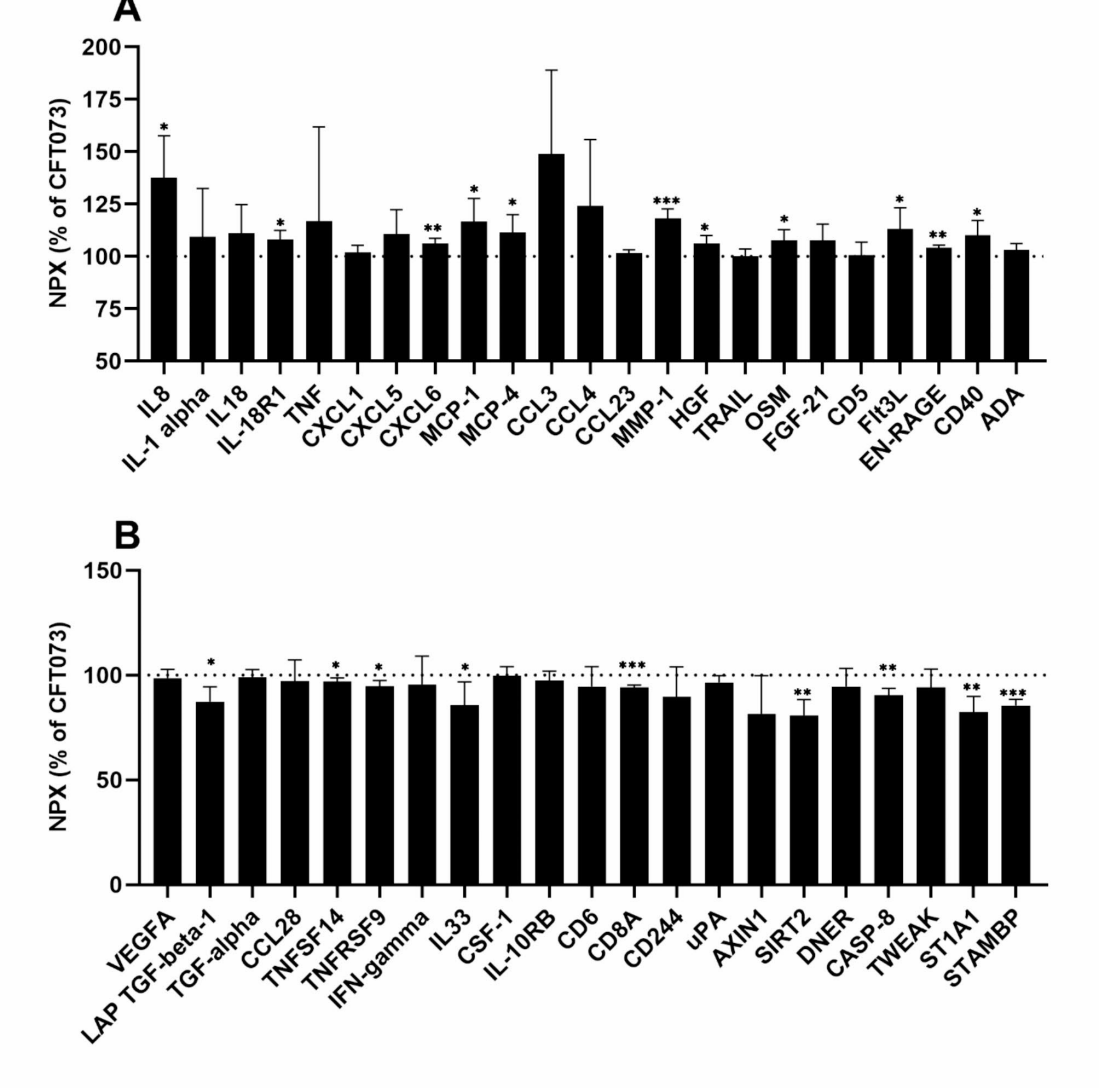
Release of inflammatory proteins from neutrophils. Neutrophils were stimulated with CFT073 at MOI 10 pre-treated with or without estradiol (300pg/ml) for 6 h followed by the analysis of 92 inflammatory proteins simultaneously using the proximity extension assay on the Proseek Multiplex inflammation panel. The protein data is presented as % normalized protein expression units (NPX) of CFT073 stimulation, where a high value corresponds to high protein concentration. Increased (**A**) or decreased (**B**) protein release in comparison to CFT7073 stimulation. The dotted line represents CFT073 stimulation. Data is shown as mean ± SEM compared to unstimulated CFT073, *n =* 4. The asterisk distinguishes statistical significance: *p < 0.05, **p < 0.01, ***p < 0.001.

To gain more knowledge on how estradiol-stimulated CFT073 affects neutrophil host response factors, we performed a targeted protein analysis of inflammation-related proteins released by neutrophils after 6 h of stimulation. We found that estradiol-stimulated CFT073 (MOI 10) induced significantly increased release of 11 proteins (IL-8, MMP-1, CXCL6, EN-RAGE, IL-18R1, MCP-1, MCP-4, HGF, OSM, Flt3L and CD40) compared to unstimulated CFT073 from neutrophils (Fig. 4A). Furthermore, the targeted protein analysis revealed that estradiol-stimulated CFT073 significantly decreased the release of 9 proteins (CD8A, STAMBP, SIRT2, CASP-8, ST1A1, LAP TGF-beta-1, TNFSF14, TNFRSF9 and IL-33) compared to unstimulated CFT073 from neutrophils (Fig. 4B).

## Discussion

UPEC mediated UTI is one of the most common bacterial infections worldwide^1^. Previous studies have underlined the protective role of estrogen for the host during a UTI^12–14^. We hypothesize that estrogen has a dual effect, both on the human host and on UPEC. We have particularly focused on the effects of estrogen on UPEC virulence. Our previous findings support this dual effect, as we have shown that estrogen alters the virulence traits of UPEC^15^. We identified a protective role of estrogen, at premenopausal levels (300pg/ml), which has been applied in this study. Estrogen alters virulence traits in UPEC, and in the present study, we have shown that these alterations modulate neutrophil responses.

We found that estradiol-stimulated CFT073 were more phagocytized by neutrophils, induced more NETs-and intracellular ROS production, but less total ROS production. Mice with compromised neutrophil function are more susceptible to UTIs and show a diminished ability to clear UPEC infections^8^. This indicates that neutrophils are essential for bacterial clearance during UTIs. Phagocytosis is generally considered to be more efficient in killing bacteria than extracellular ROS. Phagocytosis combines multiple killing mechanisms, including the production of intracellular ROS within the phagolysosome, along with the presence of other antimicrobial agents^5,6^. The increased NETs formation we observed, may also have contributed to the increased phagocytosis. NETs immobilize bacteria, making them easier to phagocytise by other neutrophils^5^. It has also been shown that intracellular ROS is positively related to phagocytosis and NETs formation^5,19^, hence linking the observed increased intracellular ROS production to phagocytosis and NETs formation. Taken together, we suggest that the estrogen mediated alterations to UPEC virulence, modulate neutrophil responses in a host beneficial way.

The NLRP3 inflammasome has been associated with the severity of a UPEC mediated UTI^20,21^and regulates the maturation and release of IL-1β and IL-18^22^. We have previously shown that CFT073 can activate the NLRP3 inflammasome to induce IL-1β release from neutrophils and bladder epithelial cells^23,24^. In the present study, we found that estradiol-stimulated CFT073 induced lower expression of genes (IL-1β, pro-Caspase-1, NLRP3 and IL-1RA) associated with the NLRP3 inflammasome. These genes are all important regulators of the innate immune response during a UTI^25–27^. Although IL-1β mRNA expression was downregulated, estradiol-stimulated CFT073 indued increased IL-1β release. We believe that this may be due to pre-synthesized pro-IL-1β, which is ready to be cleaved and released upon NLRP3 inflammasome activation in resting neutrophils^17,24,28^. Another explanation for the increased IL-1β release could be the reduced cell death (LDH release) induced by estradiol-stimulated CFT073. We have previously shown that increased neutrophil viability mediated increased IL-1β release^17^. The reduced UPEC cytotoxicity to neutrophils is an important finding, as it is in accordance with our previous findings showing that estradiol-stimulated CFT073 were less cytotoxic to *C. elegans*worms^15^. Hence, we argue that the reduced UPEC cytotoxicity is beneficial for the host and could improve the outcome during UPEC infections. The role of IL-1β in the development of UPEC-mediated UTI is contradictory. One study has found that IL-1β knockout mice are protected from UTI^29^, while other studies have shown that UPEC-induced IL-1β is an important part of the innate immune response that protects the urinary tract^26,30^. One explanation might be that the effect of IL-1β may change depending on the stage of the infection. IL-1β is essential in the early stages of infection, where it drives inflammation, recruits immune cells like neutrophils and monocytes, and aids in pathogen clearance. However, in chronic infections, sustained or excessive IL-1β production can lead to tissue damage and prolonged inflammation^31^. To summarize, we have shown that estradiol-stimulated CFT073 reduced neutrophil cytotoxicity and increased IL-1β release, which could help the host clear the infection.

One major finding in the present study was the increased release of proteins related to cell migration. IL-8 is an important neutrophil recruiting chemokine, which is present in urine during a UTI^32^. We observed that the IL-8 mRNA expression was downregulated by estradiol-stimulated CFT073. Surprisingly, IL-8 protein release was significantly increased, which was further supported by the OLINK proteomic data. Hence, the altered UPEC virulence traits induced by estradiol, mediate an increased IL-8 release. As neutrophils are essential for clearing a UTI^8^, this increase will recruit additional neutrophils to the site of infection. IL-8 has also been shown to be an essential urothelial growth factor promoting cell survival, which helps the urothelium withstand UPEC infections^33^.

Next, we evaluated the release of inflammation-related proteins from neutrophils after infection using a multiplex Olink assay. We found that estradiol-stimulated CFT073 mediated increased release of several proteins with chemotactic properties from neutrophils compared to CFT073 stimulation. In addition to IL-8, we also found that the release of the neutrophil chemotactic factor CXCL6 was increased^34^. These findings strengthen the notion that estradiol-stimulated CFT073 promotes increased neutrophil recruitment to the site of infection. Furthermore, other important chemokines, such as MCP-1 and MCP-4 were also increased after estradiol-stimulated CFT073 infection compared to CFT073 stimulation. Both MCP-1 and MCP-4 are chemokines that attract monocytes and macrophages to site of infection^35^. Macrophages in the bladder play critical roles in initiating inflammation, recruiting neutrophils, and resolving inflammation during UTIs^36,37^. Hence, an increase in chemotactic proteins specific for monocytes and macrophages could potentially promote further neutrophil migration and enhance clearance of the infection. Moreover, we also found that caspase-8 and IL-33 release was reduced by estradiol-stimulated CFT073. The reduced caspase-8 release might be a reflection of the improved cell viability mediated by estrogen-stimulated CFT073. This reduction might be beneficial for the host as caspase-8 inhibition has previously been shown to improve the outcome of bacterial infections in mice^38^. The NLRP3 associated anti-inflammatory cytokine IL-33 ^40^has been found to bind to NF-κB and reduce pro-inflammatory responses^39^. We found that several cytokines and chemokines were upregulated by estradiol-stimulated CFT073. This upregulation might be linked to the reduced autocrine effect of IL-33, which would have suppressed pro-inflammatory neutrophil responses. We also observed increased (MMP-1, IL-18R1, MMP-1, HGF, OSM, Flt3L, EN-RAGE and CD40) and decreased (LAP TGF-beta-1, TNFSF14, TNFRSF9, CD8A, SIRT2, ST1A1 and STAMBP) protein release of several inflammatory mediators that currently lack a known association to UTI. In summary, our findings demonstrate that estradiol-stimulated CFT073 promotes a pro-inflammatory milieu, which could increase the influx of additional neutrophils and monocytes to the site of infection.

This study has some limitations. We have only examined one estrogen concentration, whereas a dose-response evaluation is needed in the future. Additionally, we use a single UPEC strain (CFT073), limiting the generalizability across other UPEC strains. The mechanisms behind estrogens effect on CFT073 is still unclear and needs further evaluation.

In conclusion, our study indicates that estrogen-mediated alterations to UPEC virulence significantly modulate neutrophil responses, benefiting the host. Specifically, estradiol-stimulated CFT073 was found to reduce neutrophil cytotoxicity while increasing phagocytosis, NETs formation and the release of pro-inflammatory cytokines and chemokines. These changes likely enhance the influx of neutrophils and monocytes, creating a pro-inflammatory environment conducive to bacterial clearance. Thus, estrogen’s influence on UPEC virulence and the subsequent immune response appears to support the host in effectively combating UTIs.

## Data Availability

All data generated or analyzed during this study are included in this published article. Contact the corresponding author for data requests.
